# GRU-Based Deep Multimodal Fusion of Speech and Head-IMU Signals in Mixed Reality for Parkinson’s Disease Detection

**DOI:** 10.3390/s26010269

**Published:** 2026-01-01

**Authors:** Daria Hemmerling, Milosz Dudek, Justyna Krzywdziak, Magda Żbik, Wojciech Szecowka, Mateusz Daniol, Marek Wodzinski, Monika Rudzinska-Bar, Magdalena Wojcik-Pedziwiatr

**Affiliations:** 1 Department of Measurement and Electronics, AGH University of Krakow, 30-059 Krakow, Poland; 2 Department of Neurology, Andrzej Frycz Modrzewski Krakow University, 30-705 Krakow, Poland

**Keywords:** Parkinson’s disease, augmented reality (AR), speech analysis, voice biomarkers, inertial measurement units (IMU), head motion, multimodal data fusion, gyroscope and accelerometer signals, wearable sensing, remote digital assessment

## Abstract

Parkinson’s disease (PD) alters both speech and movement, yet most automated assessments still treat these signals separately. We examined whether combining voice with head motion improves discrimination between patients and healthy controls (HC). Synchronous measurements of acoustic and inertial signals were collected using a HoloLens 2 headset. Data were obtained from 165 participants (72 PD/93 HC), following a standardized mixed-reality (MR) protocol. We benchmarked single-modality models against fusion strategies under 5-fold stratified cross-validation. Voice alone was robust (pooled AUC ≈ 0.865), while the inertial channel alone was near chance (AUC ≈ 0.497). Fusion provided a modest but repeatable improvement: gated early-fusion achieved the highest AUC (≈0.875), cross-attention fusion was comparable (≈0.873). Gains were task-dependent. While speech-dominated tasks were already well captured by audio, tasks that embed movement benefited from complementary inertial data. Proposed MR capture proved feasible within a single session and showed that motion acts as a conditional improvement factor rather than a sole predictor. The results outline a practical path to multimodal screening and monitoring for PD, preserving the reliability of acoustic biomarkers while integrating kinematic features when they matter.

## 1. Introduction

Parkinson’s disease (PD) is a neurodegenerative disorder characterized by both motor and non-motor disturbances arising from dysfunction of basal ganglia–thalamo–cortical circuits. Deficits in movement initiation, scaling, and automatization give rise to the cardinal motor signs (bradykinesia, rigidity, tremor, postural instability) and to prominent axial manifestations such as reduced trunk–head rotation, impaired segmental coordination, and postural deformities [1,2]. Speech is frequently affected: hypokinetic dysarthria occurs in approximately 80–90% of patients and is characterized by hypophonia (reduced loudness), monopitch and monoloudness, flattened prosody, short rushes of speech, and imprecise articulation [2,3]. Mechanistically, these abnormalities reflect a combination of bradykinesia, peripheral rigidity, impaired amplitude scaling, and disrupted sensory control—including underestimation of self-produced loudness [1]. Endoscopic and imaging studies have documented glottal insufficiency and bowing or atrophy of the vocal folds, while aerodynamic analyses show reduced subglottal pressure and altered loudness strategies (Lombard effect), pointing to combined respiratory–laryngeal dysfunction [4,5].

Motor scaling deficits in PD extend across both orofacial and axial effectors. Axial symptoms include pronounced rigidity, reduced range of trunk–head rotation, *en bloc* coupling of head and trunk during turning, and an increased risk of falls due to decreased trunk mobility. Postural deformities such as camptocormia, antecollis, and Pisa syndrome further disrupt head orientation. Although head tremor is not a cardinal PD sign, subtle oscillatory or jerky head movements may appear and require differentiation from essential or dystonic tremor. Taken together, simultaneous measurement of speech acoustics and head kinematics has the potential to capture complementary pathophysiological markers: (i) a respiratory–laryngeal component (hypoadduction, reduced subglottal pressure, dysprosody) and (ii) an axial component (restricted range of motion, reduced velocity, decoupling of head–trunk movement, increased stiffness) [1,4]. Clinically and metrologically relevant metrics span head rotation/tilt ranges, angular velocity and jerk, and tremor-related spectral power (3–7 Hz), analyzed alongside acoustic features such as intensity, F0 variability, laryngeal tremor indices, and temporal discontinuities.

These clinical observations motivate the development of digital biomarkers that quantify PD-related changes in speech and movement in a scalable, objective manner. Voice is a particularly mature source of digital markers: classical signal processing and modern representation-learning approaches have demonstrated robust performance for PD detection and monitoring based on sustained phonation, reading, diadochokinetic tasks, and spontaneous speech [6,7]. At the same time, inertial measurement units (IMUs) placed on the trunk, limbs, or head capture kinematic signatures of gait, posture, tremor, and balance that are central to PD pathophysiology but often underrepresented in purely audio-based pipelines [8,9]. From a multimodal perspective, fused analysis of voice and movement is attractive: voice encodes fine-grained motor-speech control, whereas IMU-based kinematics provide context about axial and whole-body motor function. A concise overview of representative smartphone-, smartwatch-, and MR/AR-based digital-biomarker studies in PD is given in Table 1,which highlights both the rapid progress in sensor-based assessment and the relative lack of tightly synchronized voice–head-IMU pipelines in mixed reality. Mixed and augmented reality (MR/AR) head-mounted displays (HMDs) provide a natural platform for such multimodal assessment. Devices like Microsoft HoloLens 2 (HL2) combine standardized, programmable task elicitation with precise on-device sensing (microphones, cameras, IMUs), enabling reproducible measurements under controlled stimuli while preserving ecological validity [8,9,10]. Validation studies have shown that HL2 can yield accurate and reliable kinematic readouts for gait, balance, and upper-limb tasks, supporting its use in clinical assessments and longitudinal follow-up [8,9,11]. The headset-mounted microphone array offers standardized mouth–microphone geometry, while the rigidly attached IMU follows head motion without body–sensor displacement artifacts. This combination makes MR/AR HMDs a promising testbed for synchronized audio–kinematic biomarkers in PD.

Over the past two years our group has systematically explored MR-based assessments in PD. We have shown that (i) speech collected in an MR setting supports PD screening and task-specific analysis of acoustic and linguistic features [6], (ii) MR implementations of gait tests (e.g., Timed Up-and-Go) yield discriminative spatiotemporal characteristics between PD and healthy controls [18], and (iii) MR can be used to deliver multicomponent motor–cognitive and eye-tracking tasks on HoloLens 2 [20,21]. In these earlier studies, however, speech and motion channels were analyzed separately rather than fused within a synchronized MR pipeline, and the potential of concurrent voice–head kinematics for PD assessment remained unexplored.

In this work, we introduce DiagNeuro, an AR system for simultaneous acquisition of voice and head-IMU signals during interactive, standardized tasks with time-locked synchronization and a unified processing stack. We develop and evaluate a set of deep-learning models that operate on short, synchronized audio–IMU episodes to discriminate PD from healthy controls. Our contributions are threefold: (i) an MR acquisition architecture that ensures sample-level alignment between audio and head kinematics under guided protocols; (ii) a multimodal learning pipeline with early, intermediate, and gated fusion strategies complemented by probability calibration; and (iii) an analysis of how such MR-based fusion could translate to mobile deployments (e.g., smartphone-grade microphones and IMUs) while preserving task standardization. We specifically test the hypotheses that (H1) multimodal models fusing voice and head-IMU features outperform voice-only and IMU-only baselines for PD vs. HC discrimination; (H2) the incremental benefit of fusion is task-dependent, with larger gains in tasks that more strongly engage movement; and (H3) synchronized MR acquisition is feasible in a clinical setting and yields multimodal markers that align with established PD pathophysiology.

From a modeling perspective, we cast this study as a supervised binary classification problem. The input is a short, synchronized voice–IMU episode recorded during one of five standardized mixed-reality speech tasks (T02–T06), and the target label is the diagnostic group y∈{PD,HC}. We analyze both a pooled configuration, where episodes from all tasks are combined to train a single classifier, and task-wise models that are fit and evaluated separately for each task. This formulation links the clinical questions to the technical pipeline: Section 2 details how episodes and clinical variables are stored in the database, and Section 3 describes the preprocessing and multimodal fusion architectures used to learn from these episode-level records.

## 2. Database and Data Acquisition

### 2.1. Participants and Clinical Data

All participants were recruited prospectively from a single-center cohort. Individuals with Parkinson’s disease fulfilled Movement Disorder Society (MDS) diagnostic criteria and were clinically able to comply with mixed-reality (MR) task instructions and headset use. Exclusion criteria included other major neurological disorders, unstable psychiatric disease, decompensated cardiopulmonary conditions, or acute intercurrent illness. Healthy controls (HC) reported no neurological disease and had normal or corrected-to-normal vision and hearing. All Parkinson’s disease participants were evaluated in the medication **ON** state (i.e., while taking their regular dopaminergic therapy) at the time of data acquisition. The study was approved by the local Institutional Review Board/Ethics Committee (protocol ID: KBKA/40/O/2022, 14.07.2022) and was conducted in accordance with the Declaration of Helsinki and GDPR. All participants provided *written informed consent* prior to enrollment. Sessions were conducted under supervision of trained staff using Microsoft HoloLens 2. Participants remained seated for speech tasks, were instructed to maintain a neutral head posture, and could pause or withdraw at any time. The final dataset comprised 93 healthy controls (464 sessions) and 72 Parkinson’s disease participants (358 sessions). The exact numbers are shown in Table 2.

Clinical characterization of the PD cohort followed routine practice. To reflect the distribution of complete multimodal episodes across tasks, for each task, we report PD summaries computed on sampled task-sized subsets drawn from the PD cohort (Table 3). The following scales were used:

*MDS–UPDRS* (Movement Disorder Society Unified Parkinson’s Disease Rating Scale) is a widely used reference composite clinical scale for PD severity and impairment (often treated as a practical “gold-standard” outcome in clinical research and in validation studies of digital biomarkers) [22,23,24,25,26]. It comprises four parts with item-level ratings (typically 0–4 per item; higher = worse) [23]:

- Part I (Non-motor experiences of daily living): neuropsychiatric, sleep, autonomic, pain, fatigue—patient/clinician reported. Summed score reflects non-motor burden.
- Part II (Motor experiences of daily living): patient-reported motor impact in everyday activities (speech, handwriting, dressing, hygiene, turning in bed, walking/balance).
- Part III (Motor examination): clinician-rated cardinal motor signs (rigidity, bradykinesia, tremor, posture, gait, postural stability, facial expression, speech intelligibility). This is the primary snapshot of current motor impairment; higher values indicate more severe motor dysfunction.
- Part IV (Motor complications): dyskinesias and motor fluctuations (duration, functional impact). Elevated scores indicate treatment-related complications.

We also report the MDS-UPDRS total (sum of Parts I–IV when available) as an overall severity index UPDRS-T. Interpretatively, Part III correlates with examiner-observed motor deficits, Parts I–II reflect patient-experienced symptom burden, and Part IV captures treatment complications—together providing a multidimensional phenotype. *Hoehn & Yahr (H&Y)* is a succinct *stage* classifier (0–5; higher = more advanced disease) emphasizing laterality, balance, and postural instability [27]. In addition to the original five-stage description, modified H&Y variants (including intermediate stages such as 1.5 and 2.5) are widely used in clinical research and are discussed in the MDS Task Force report and clinical staging summaries [28,29]. Typical anchors: Stage 1 (unilateral signs), Stage 2 (bilateral without postural instability), Stage 3 (postural instability but physically independent), Stage 4 (severe disability; still able to walk/stand unassisted), Stage 5 (wheelchair/bedbound unless aided). H&Y is widely used for cohort stratification and prognosis.

*Schwab & England (S&E)* indexes functional independence in activities of daily living from 100% (normal; completely independent) down to 0% (vegetative) [30]. Operationally, S&E is recorded as a percentage estimate of speed and independence in ADL (higher = better function), and its scoring anchors are available in standardized clinical data element repositories and clinical guidance resources [31,32]. Decrements reflect increasing need for assistance and reduced efficiency (e.g., 70–80%: independent but slow; 50–60%: needs occasional to frequent help). S&E complements H&Y with a continuous functional perspective.

*MSDSRS* (Motor Speech Disorder Severity Rating Scale) is a clinician-rated global index of motor-speech severity. In neurodegenerative motor-speech phenotyping, MSDSR is commonly operationalized as a 10-point clinician rating (e.g., 1 = nonvocal; 10 = normal), and has been used together with other perceptual motor-speech measures in prior work [33,34]. It reflects perceptual severity across speech subsystems (articulation, phonation/voice quality, prosody/rate, resonance) and overall communicative effectiveness; higher scores denote better motor-speech function. The characteristic hypokinetic dysarthria profile in PD (e.g., reduced loudness, monopitch/monoloudness, short rushes of speech, imprecise consonants) is described in clinical reviews [2].

*VHI* (Voice Handicap Index) is a patient-reported outcome quantifying the perceived impact of voice problems across three subscales—*Functional*, *Physical*, and *Emotional* [35]. Items are scored 0–4 and summed (higher = greater perceived handicap). VHI complements examiner ratings by capturing subjective burden, which may be discordant with objective measures but is clinically salient.

MDS–UPDRS provides the canonical neurological anchor (signs, daily impact, and treatment complications) [23,36]. H&Y and S&E add global stage and functional independence. MSDSRS and VHI bridge to the speech domain, quantifying both clinician-observed motor-speech impairment and patient-perceived voice limitations. This combination enables the following: (i) clinical interpretability of model output in relation to motor stage and function; (ii) alignment of acoustic/IMU markers with examiner-rated dysarthria; and (iii) assessment of patient-centered outcomes relevant to communication. For HC, formal neurological scales were not administered within this protocol; instead, we report task-wise counts and demographic composition, together with an explicit note of neurological health (no neurological diagnosis, no dopaminergic therapy), mirroring the PD presentation while avoiding inference on unmeasured scales (Table 3).

### 2.2. MR System and Speech/IMU Acquisition

**Figure 1:**
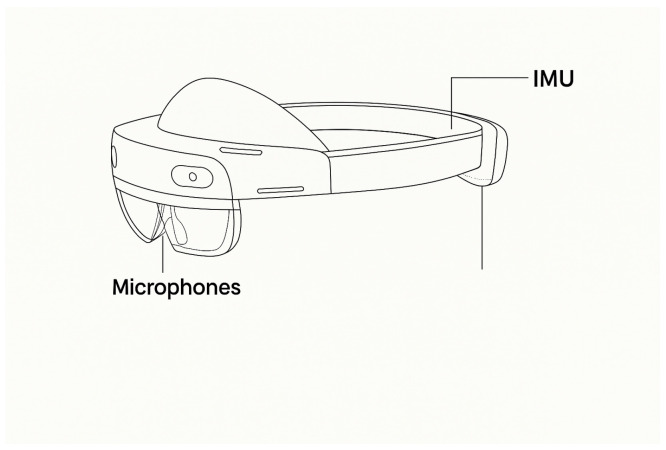
Approximate placement of sensing elements on Microsoft HoloLens 2. The IMU package (accelerometer, gyroscope; with co-located magnetometer on the device) is embedded in the visor near the centerline and rigidly follows head motion. The multi-microphone array is distributed along the front/side rim of the visor and temple sections, providing forward-facing, beamformed audio capture.

DiagNeuro is a self-contained mixed-reality pipeline that records speech acoustics and head kinematics concurrently on a Microsoft HoloLens 2, operating fully offline during patient sessions. Two sensor families are used: a five-microphone array for audio capture and an inertial measurement unit (IMU) with tri-axial accelerometer and gyroscope. Access to synchronized sensor streams and device-clock timestamps is provided via HoloLens 2 Research Mode, which exposes raw IMU and environmental cameras to research applications while restricting raw eye-camera imagery for privacy [37,38]. Figure 1 shows the on-device locations of the inertial sensors and microphones on Microsoft HoloLens 2 used in this study.

The inertial measurement unit (IMU; tri-axial accelerometer and tri-axial gyroscope, with co-located magnetometer on the device) is integrated in the front visor module near the headset midline, i.e., rigidly attached to the user’s head. This placement ensures that IMU axes follow the headset’s local coordinate frame, capturing head motion without body–sensor displacement artifacts. The built-in beamforming microphone array is distributed along the lower rim of the visor and the adjacent temple sections, facing forward towards the talker’s mouth while remaining offset from the airflow path. This geometry provides near-field speech capture with improved rejection of environmental noise and enables synchronized, co-registered audio–IMU recordings for multimodal fusion.

Audio is captured as multichannel recordings from the onboard microphone array at 48 kHz with floating-point samples, preserving spatial information for optional post hoc beamforming and noise-robust feature extraction; channels are time-aligned by the on-device mixer [39]. Head kinematics are logged continuously as specific force and angular velocity in the headset reference frame. Before the first speech task, a short calibration segment (3–5 s of silence and stillness) is recorded to estimate ambient noise, accelerometer bias relative to gravity, and gyroscope drift. These estimates are then used for gravity compensation, detrending, and quality control of motion-related artifacts in the speech signal. All streams (audio, IMU, and task events) are stamped by a common device clock, serialized with metadata and integrity hashes, and exported only after session completion in encrypted containers approved for hospital environments. The choice of HoloLens 2 is supported by reports demonstrating concurrent validity and test–retest reliability of mixed-reality measurements in Parkinson’s disease cohorts [9,17,40]. All signals were acquired on Microsoft HoloLens 2: speech was recorded at 48 kHz, while head-IMU data were captured via the Research Mode IMU streams (accelerometer: 93 samples/frame; gyroscope: 315 samples/frame; effective sampling rates ≈1.1 kHz and ≈3.8 kHz, respectively). Any resampling/downsampling was preceded by an anti-aliasing low-pass filter (cutoff $<f_{s}/2$), ensuring compliance with the Shannon–Nyquist criterion.

### 2.3. Task Battery and MR Interface

**Figure 2:**
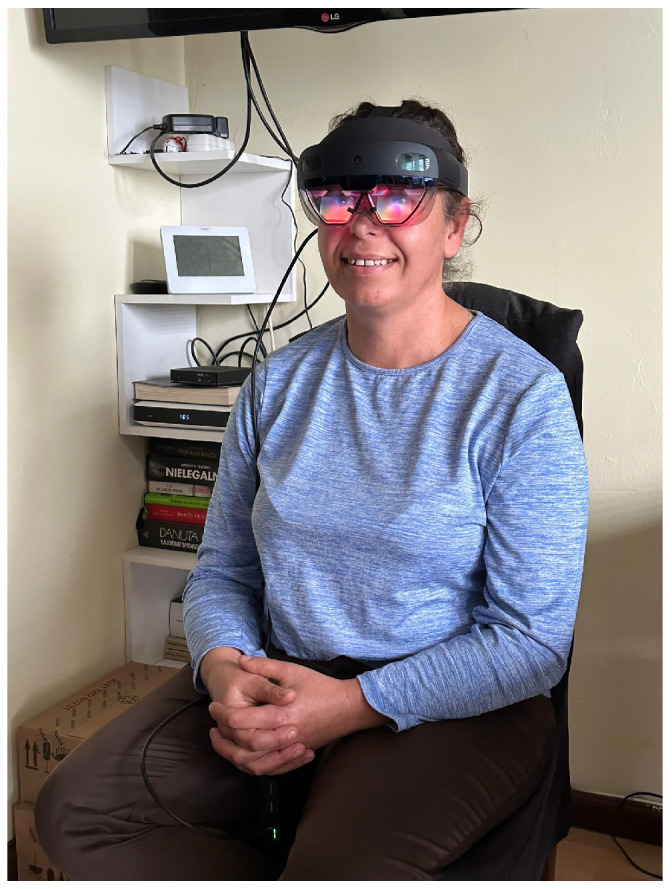
Representative healthy control participant (female, 58 years) wearing HoloLens 2 during recording. The photograph illustrates typical headset placement in our study and the relative orientation between mouth, on-device microphone array, and embedded IMU. Facial details are de-identified; publication consent was obtained.

The protocol targets hypokinetic dysarthria and related speech–motor phenomena in Parkinson’s disease while simultaneously tracking head micro-movements. Tasks are presented as holographic overlays with spoken prompts; participants advance with gaze or short voice commands (*Start*, *Repeat*, *Next*). Throughout the block (approximately 15–20 min), participants are instructed to maintain a neutral head posture what can be seen on Figure 2. All head motion is recorded for kinematic analysis and for modeling motion-induced acoustic variability.

**Figure 3:**
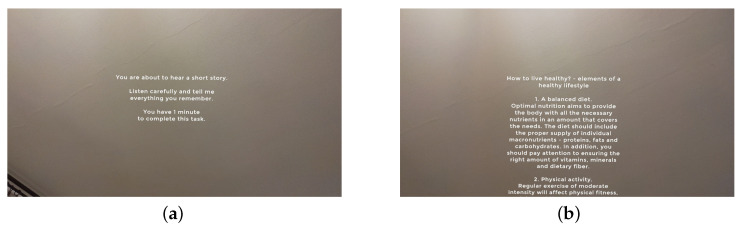
**Example MR screens during the speech battery:** (**a**) screen with task title and concise instructions; navigation via short voice commands (“Start”, “Repeat”, “Next”); (**b**) task view with the same standardized layout enabling repeatable prompts and responses. The figure includes the key UI elements (task title, instruction area, and there is a voice-command navigation) and to clarify the correspondence between panels and protocol steps. The standardized holographic interface supports consistent timing and protocol adherence while audio and head-IMU signals are recorded concurrently and synchronized to device-clock events.

The battery comprises the following:

1. Picture description (spontaneous speech) (T02): free description of a static scene for 30 s, enabling measurement of speaking rate, pause structure, fundamental frequency variability, and natural loudness modulation with minimal lexical constraint.
2. Daily activity report (T03): 60 s spontaneous monologue describing the participant’s typical day (morning–evening routines and tasks). The task elicits ecologically valid connected speech with minimal lexical scaffolding and supports analysis of speaking/articulation rate, pause fraction, prosodic variability, and disfluency counts.
3. Story recall (T04): prompted recall of a short, three-part vignette (healthy eating, physical activity, healthy sleep) over 45–60 s. Participants first receive the story and then retell it, enabling quantification of temporal fluency, prosodic dynamics, lexical diversity, and content-unit recall under mild cognitive–linguistic load. Example MR screen for this task can be seen on Figure 3.
4. Syllable repetition (diadochokinesis) (T05): AMR on /pa/, /ta/, /ka/ and SMR on /pataka/ in 10 s trials (two per pattern). Extracted metrics include syllable rate, regularity, initiation latency, and temporal stability, which show characteristic differences in Parkinson’s disease [1,2,3].
5. Sustained vowel phonation (T06): sustained /a/ at comfortable loudness for 10 s (three trials), followed by an optional loud condition. This task yields robust phonatory features (fundamental frequency, intensity, jitter, shimmer, harmonic-to-noise ratio) that are sensitive to hypokinetic dysarthria [1,2].

Across all tasks, the head-mounted IMU runs continuously. Kinematic metrics include head rotation and tilt ranges, angular velocity and jerk, and spectral power within tremor-relevant bands (approximately 3–7 Hz). These trajectories are analyzed jointly with acoustic features to quantify voice–head coupling (for example, cross-correlation analyses) and to regress motion confounds where appropriate. Together, the tasks operationalize the core dimensions of hypokinetic dysarthria—reduced loudness and pitch variability, timing irregularities, and articulatory underscaling—within a standardized MR environment [1,2,9,40].

### 2.4. Dataset Structure and Modalities

**Figure 4:**
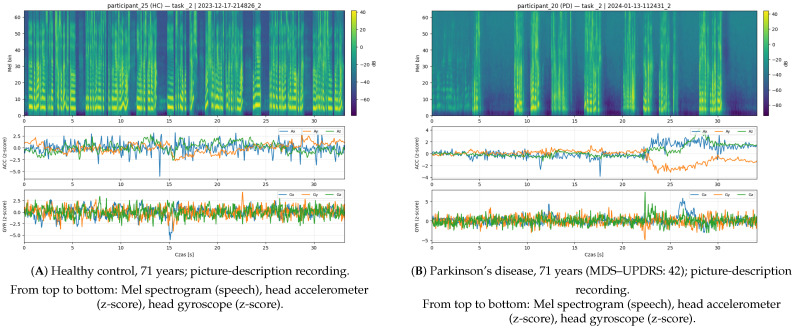
Comparison of two picture-description recordings: panel (**A**) healthy older adult vs. panel (**B**) individual with Parkinson’s disease.

Across both cohorts, the number of sessions per participant was balanced and comparable between groups. Table 3 summarizes the number of participants, recorded sessions, and available modalities (audio, accelerometer, and gyroscope) for PD and HC. “Complete” refers to sessions containing all three synchronized modalities. Example processed recordings for both healthy older adult and participant with Parkinson’s disease can be seen on Figure 4.

Each participant contributed between one and six tasks per session, depending on the experimental protocol completion rate. Task-wise availability of multimodal recordings per group is detailed in Table 3. The sustained vowel (T06) and diadochokinetic repetition (T05) tasks show the highest rate of complete multimodal recordings, suggesting robust synchronization between the audio and inertial sensors.

The global inventory shown in Table 3 presents near-parity in availability across audio and inertial channels, with a high fraction of tri-modal sessions (≈47%). This balance supports robust multimodal modeling without systematic bias toward a single sensing modality.

In the final analysis dataset, each recording is stored as a task-level “episode” comprising (i) three time-synchronized sensor streams (speech waveform, tri-axial accelerometer, tri-axial gyroscope) and (ii) an associated metadata record. The sensor streams are referenced by file paths (wav_path, acc_path, gyr_path), while the metadata includes a unique participant identifier, diagnostic group (PD/HC), task identifier (T02–T06), session index, and available clinical scores (age, sex, MDS–UPDRS, H&Y, S&E, MSDSRS, VHI where available). This relational organization defines the database used in the subsequent modeling steps (Section 3) and ensures that episodes can be grouped by subject, task, or clinical strata for subject-aware cross-validation.

## 3. Methodology

### 3.1. Overall Workflow

**Figure 5:**
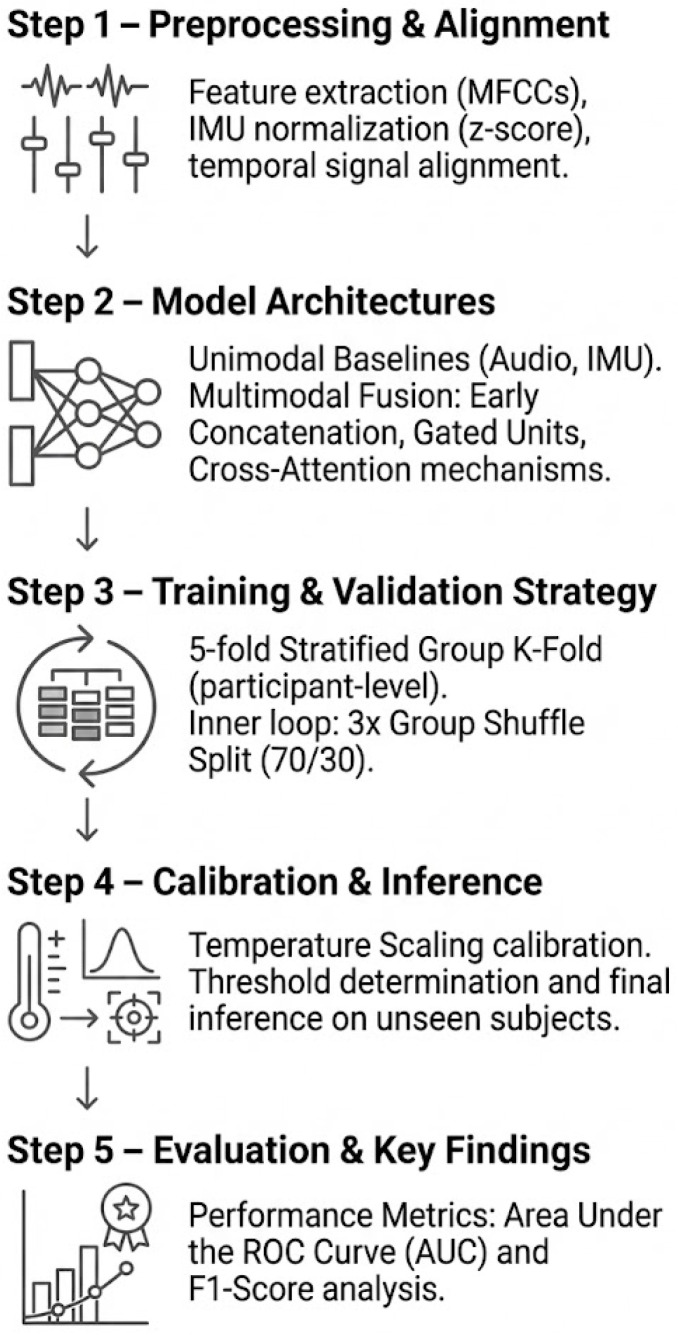
Study plan and processing pipeline: MR acquisition (voice + head IMU) during standardized tasks; preprocessing and synchronization; model variants (*audio_only*, *imu_only*, *early_concat*, *mid_xattn*, *gated_early*); training and evaluation (cross-validation, calibration, AUC/F_1_ metrics); key result: fusion modestly outperforms audio-only, with larger gains in movement-engaging tasks.

Figure 5 summarizes the modeling workflow used in this study. Briefly, we (1) acquire synchronized voice and head-IMU data in mixed reality using the DiagNeuro headset protocol; (2) preprocess and temporally align the signals (log mel-spectrograms for audio, normalized six-axis IMU at 100 Hz) into fixed-length crops; (3) instantiate a set of candidate architectures (audio_only, imu_only, early_concat, mid_xattn, gated_early); (4) train and evaluate these models under stratified, subject-aware cross-validation with probability calibration; and (5) compare pooled and task-wise performance, focusing on whether fusion yields a consistent gain over audio-only baselines, particularly in movement-engaging tasks.

### 3.2. Signal Preprocessing and Temporal Alignment

Audio waveforms are loaded at 16 kHz, converted to mono, and peak-normalized to unit amplitude. We extract log mel-spectrograms with $F=N_{\mathrm{mels}}=56$, $N_{\mathrm{FFT}}=1024$, and hop length =320 samples (≈20 ms). For numerical stability, we add a $10^{-10}$ floor prior to log(·).

For accelerometer and gyroscope streams, we identify the time column (e.g., time, timestamp, ticks) via heuristic matching and select three numeric axes corresponding to {*x,y,z*}. Missing samples are forward/back-filled, time is re-based to start at zero, and each stream is linearly interpolated and resampled to 100 Hz. The resulting 6-channel IMU vector (ACC_*x,y,z*_, GYR_*x,y,z*_) is per-channel median-centered and standardized by its standard deviation (with 10^−6^ floor).

We operate on synchronized audio–IMU windows of 15 s. If a session exceeds 15 s, we sample a random crop during training; at inference we apply test-time augmentation (TTA) with K=3 independent random crops. If a session is shorter than 15 s, we use the full duration. Audio and IMU are time-aligned and truncated to the common overlap length *T* (minimum of the two modalities). With the chosen parameters, a 15 s crop yields ≈750 mel frames and 1500 IMU steps.

To avoid implicit imputation artifacts in multimodal learning, sessions with incomplete modality availability are excluded from multimodal training/evaluation (see Table 2). Unimodal baselines (audio_only, imu_only) are trained and evaluated on the corresponding single-modality subsets. In practice, after synchronization the model consumes paired sequences truncated to the shared overlap; hence the effective sequence length *T* is determined by the minimum available duration across modalities within each crop.

### 3.3. Model Architecture and Multimodal Fusion

All variants share the same backbone structure: (i) modality-specific encoders producing per-timestep embeddings, (ii) an optional fusion block, (iii) a temporal model, and (iv) a classification head producing a binary logit for {HC,PD}. Importantly, the temporal model and head are matched across variants so that performance differences can be attributed to the fusion mechanism rather than increased classifier capacity.

Let $X^{\left(a\right)}\in R^{T\times F}$ denote the log-mel sequence and $X^{\left(i\right)}\in R^{T\times 6}$ the IMU sequence (after temporal alignment and truncation to the shared overlap). Both are projected to a common embedding dimension H=96,

$$A={\left\{a_{t}\right\}}_{t=1}^{T},\;I={\left\{i_{t}\right\}}_{t=1}^{T},\;a_{t},i_{t}\in R^{H}.$$

For audio, we apply two 1D temporal convolutions (kernel size 5, ReLU) to map $R^{T\times 56}\;\to \;R^{T\times 96}$. Concretely, the Conv1D stack uses channel mapping 56→96→96 with padding chosen to preserve the temporal length *T*. For IMU, we apply a per-timestep MLP (Linear–ReLU–Linear) mapping $R^{6}\;\to \;R^{96}$. We implement this as 6→96→96 applied independently at each timestep. A sinusoidal positional encoding $\mathrm{PE}\in R^{T\times H}$ is added to each modality embedding.

Because audio and IMU are sampled at different nominal rates (mel hop ≈20 ms $\Rightarrow T_{a}\approx 750$ frames per 15 s, IMU resampled at 100 Hz $\Rightarrow T_{i}\approx 1500$ samples per 15 s), we align modalities on the *mel timestamp grid*: after resampling IMU to 100 Hz, we linearly interpolate the 6-axis IMU stream onto the mel-frame timestamps, yielding $X^{\left(i\right)}\in R^{T\times 6}$ with the same *T* as $X^{\left(a\right)}$. Thus, for a synchronized 15 s crop the end-to-end tensor flow is raw crop $\to X^{\left(a\right)}\in R^{T\times 56}$ with T≈750 and IMU → interpolation onto mel timestamps $X^{\left(i\right)}\in R^{T\times 6}$; encoders $\to A,I\in R^{T\times 96}$; fusion $\to Z\in R^{T\times D}$; BiGRU $\to H\in R^{T\times 2H_{\mathrm{gru}}}$; mean pooling $\to \bar{h}\in R^{2H_{\mathrm{gru}}}$; MLP head → scalar logit.

We evaluate the following fusion strategies: (i) audio_only: Z=A; (ii) imu_only: Z=I; (iii) early_concat: $z_{t}=\left[a_{t}\;∥\;i_{t}\right]\in R^{2H}$; (iv) gated_early: an early-concatenation representation modulated by a learnable per-timestep gate,

$$z_{t}=\left[a_{t}\;∥\;i_{t}\right],\;g_{t}=\sigma \left(\mathrm{MLP}\left(z_{t}\right)\right),\;{\hat{z}}_{t}=g_{t}⊙z_{t};$$

the gate MLP is 2H→H→2H with a sigmoid output. (v) mid_xattn: a mid-level cross-attention block where audio queries IMU (keys/values) to obtain an IMU-conditioned audio representation,

$$\tilde{A}=\mathrm{LN}\;\left(A+\mathrm{MHA}(Q=A,K=\phi (I),V=\phi (I))\right),$$

and we set $Z=\tilde{A}\in R^{T\times H}$ as the fused sequence passed to the shared temporal backbone. All fusion modules are trained end-to-end jointly with the encoders and temporal backbone.

Given the fused per-timestep sequence ${\left\{z_{t}\right\}}_{t=1}^{T}$ (dimension D∈{H,2H} depending on the variant), we model temporal dependencies using a bidirectional GRU:

$${\left\{h_{t}\right\}}_{t=1}^{T}=\mathrm{BiGRU}\left({\left\{z_{t}\right\}}_{t=1}^{T}\right),$$

followed by mean pooling $\bar{h}=\frac{1}{T}\sum_{t=1}^{T}h_{t}$ and a two-layer MLP head with dropout producing a single logit. The same BiGRU and head configuration is used for all unimodal and multimodal variants. The BiGRU uses 1 layer with hidden size $H_{\mathrm{gru}}$ = 128 per direction (hence $h_{t}\in R^{2H_{\mathrm{gru}}}$). The MLP head is $2H_{\mathrm{gru}}$(=256) →128→1 with ReLU activations and dropout p=0.20.

### 3.4. Training, Validation, and Calibration Protocol

All models are trained end-to-end from random initialization. We do not employ external pretrained backbones, nor any fine-tuning strategy in this study; this isolates the effect of the fusion mechanisms and temporal modeling under a controlled-capacity regime.

We use an external 5-fold StratifiedGroupKFold split by participant identity to prevent subject leakage across folds. Within each training fold, we perform 3× GroupShuffleSplit (train/validation = 70/30, grouped by participant) to obtain internal validation sets used only for early stopping and post hoc calibration (not for architectural re-tuning). Test metrics for a fold are obtained by ensembling the three independent repetitions (probabilities averaged after calibration). Thus, hyperparameters are held constant across outer folds; the inner splits are used strictly for early stopping, calibration, and threshold selection rather than per-fold hyperparameter search.

We optimize class-weighted cross-entropy with AdamW. Early stopping is based on a smoothed validation AUC computed as the mean of the last three epochs, with patience =4. We apply gradient clipping for numerical stability. All fixed training hyperparameters are learning rate $10^{-3}$, weight decay $10^{-2}$, batch size 32, maximum epochs 50, and gradient clipping max_norm=1.0; these are reported explicitly in Table 4.

At test time, we apply TTA with K=3 random 15 s crops per sample; logits are averaged across crops prior to calibration.

To obtain well-calibrated probabilities and a stable operating point, we apply temperature scaling on the internal validation set and select an operating threshold $t^{★}$ on the calibrated probabilities. We select $t^{★}$ by maximizing validation F1 on the calibrated probabilities within the guard-rail interval. To prevent degenerate calibration on small validation sets, we enforce guard-rails:

$$T\in \left[0.300,3.000\right],\;t^{★}\in \left[0.200,0.800\right],$$

and for $N_{\mathrm{val}}<20$ we use the conservative fallback T=1.000, $t^{★}=0.500$. This regime is frequently triggered in task-wise evaluation (typical $N_{\mathrm{val}}\approx 15-21$), and any fallback or clipping is logged as red_flags for auditability.

Calibration statistics are reported for transparency. Across pooled (ALL) experiments, the average calibration temperature and operating threshold were as follows: early_concat: T=1.023±0.466, $t^{★}=0.395\pm 0.174$; gated_early: T=1.143±0.535, $t^{★}=0.471\pm 0.166$; mid_xattn: T=1.015±0.401, $t^{★}=0.467\pm 0.183$; audio_only: T=1.126±0.502, $t^{★}=0.397\pm 0.198$. For imu_only, calibration frequently saturates at the guard-rails (e.g., *T* clipped to 3.0), consistent with near-chance validation AUC in the pooled setting and preventing unstable post hoc probability scaling.

### 3.5. Metrics

Primary metric is ROC AUC computed on calibrated probabilities. We additionally report F1, precision, and recall at the selected threshold. We also report Accuracy at the selected operating point, to complement the threshold-dependent metrics and facilitate comparison with prior studies. For interpretability, we compute per-task metrics by grouping test episodes by task_id. Per-task summaries are reported for AUC (threshold-free) as well as for F1 and Accuracy (threshold-dependent). Fold-level scores are summarized by arithmetic means across outer folds. Unless stated otherwise, the operating threshold is selected on inner validation (per outer fold) and then applied to the corresponding held-out test fold.

### 3.6. Implementation Details

The pipeline is implemented in PyTorch 2.8.0. Audio I/O prioritizes torchaudio and falls back to soundfile or scipy as needed. Hidden size is H=96, and for the cross-attention variant we use multi-head attention with 4 heads ($H=96\Rightarrow d_{\mathrm{head}}=24$). Dropout is applied in a component-specific manner (e.g., p=0.20 in the classifier head; p=0.10 in gating/attention modules where applicable). All random draws (crop positions, splits, weight initialization) are seeded for reproducibility. All experiments ensure subject-wise separation between training, validation, and testing partitions via group-aware splitting.

## 4. Results of Fusion and Interpretation

We first assess whether fusion improves performance on average across heterogeneous tasks, and we quantify the AUC–F_1_ trade-off after calibration. Table 5 reports pooled means over five outer folds, including accuracy, precision, and recall. gated_early attains the highest mean AUC (0.875) and improves F_1_ versus mid_xattn (0.771 vs. 0.763), while audio_only still yields the highest mean F_1_ overall (0.785). In terms of accuracy, gated_early is also best on average (0.806), closely followed by audio_only (0.802), indicating that fusion yields a modest but consistent gain at the chosen operating point. The imu_only baseline remains near-chance AUC (0.497) with recall skew (0.983), underscoring limited standalone discriminative power of IMU and its role as a modulator when fused. Notably, mid_xattn exhibits higher recall (0.812) at the expense of precision (0.729), suggesting a more liberal decision boundary relative to gated_early (precision 0.829, recall 0.730) under the selected thresholds.

**Figure 6:**
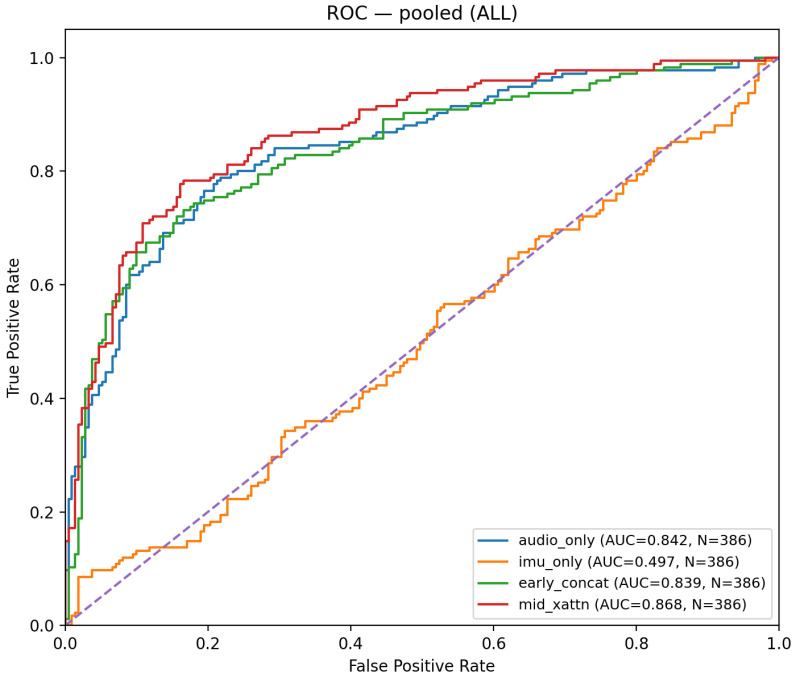
Pooled ROC curves (pooled across folds) for gated_early, mid_xattn, audio_only, early_concat, and imu_only.

Figure 6 visualizes pooled ROC curves for the main model variants, confirming the ranking observed in Table 5 and illustrating the operating regimes where fusion provides the largest gain (e.g., moderate false-positive rates where gated_early dominates).

We next analyze task-dependent effects to reveal when IMU contributes the most. As shown in Table 6, gated_early is top on **T03** (AUC 0.936) and **T06** (AUC 0.854), early_concat ranks best on **T02** (AUC 0.854), and audio_only ranks best on **T04** (AUC 0.926) and **T05** (AUC 0.805). Two qualitative patterns follow (i) tasks with strong acoustic signatures (T04, T05) are saturated by audio, leaving limited headroom for IMU; (ii) tasks requiring fine-grained movement context (T03, T06) benefit from injecting IMU at the representation level with an explicit gate, while T02 favors simple concatenation.

**Figure 7:**
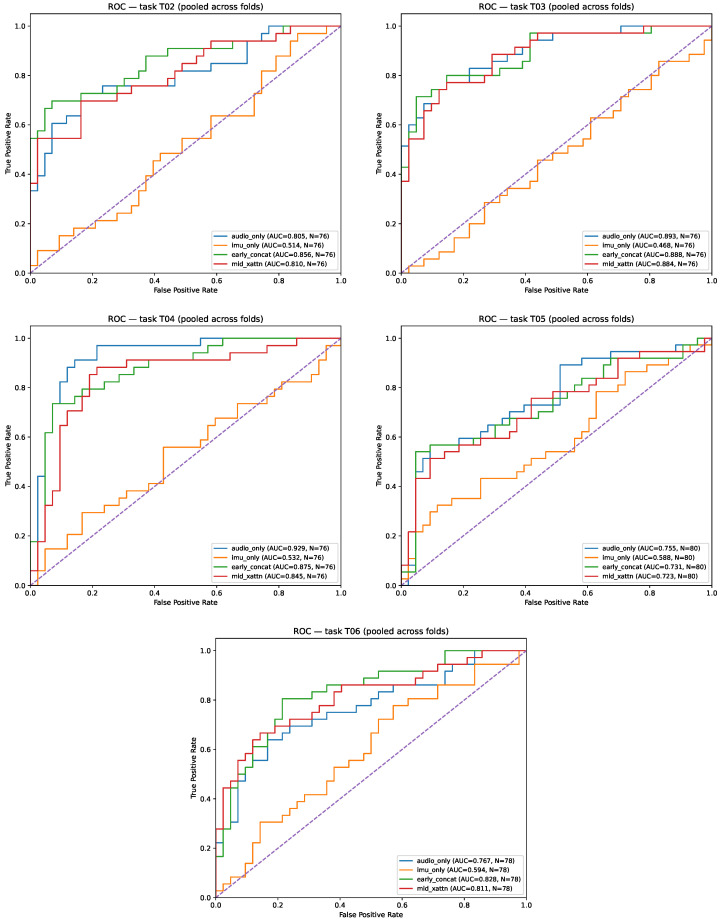
Per-task ROC curves (mean over outer folds) for tasks T02–T06. Each panel compares gated_early, mid_xattn, audio_only, early_concat, and imu_only. Audio_only uses speech only; imu_only uses head-IMU only; fusion models (early_concat, mid_xattn, gated_early) use synchronized speech + IMU streams.

Figure 7 provides per-task ROC curves (one panel per task), showing that task heterogeneity is substantial: T04 and T05 exhibit strong separability for audio_only, whereas T03 (and, to a lesser extent, T06) benefits from IMU-conditioned fusion, consistent with the per-task AUC maxima in Table 6. The per-task ROC curves (Figure 7) further highlight this heterogeneity.

To complement threshold-free AUC with operating-point behavior, Table 7 and Table 8 report per-task F1 and accuracy, respectively. These results reveal that the method with the best AUC per task does not always maximize F1/accuracy on the same task. For example, on T06 gated_early has the highest AUC (0.854) but early_concat achieves a substantially higher F1 (0.761), indicating that the globally selected threshold (optimized on inner validation) may be better aligned with early_concat for that task’s score distribution. Similarly, on T02 early_concat is best in AUC (0.854) and F1 (0.722), whereas audio_only attains the best accuracy (0.790), consistent with a threshold-dependent trade-off between precision/recall and overall correctness.

Table 9 summarizes the best method per task (selected by per-task AUC) and reports the corresponding F1/Accuracy/Precision/Recall at the operating point. The shift from early_concat to gated_early on T03 and T06 indicates that learning when and how much to trust IMU, rather than always concatenating, can improve separability while maintaining stable temporal modeling. More broadly, we observe a consistent hierarchy: (a) IMU-only remains insufficient across tasks (low AUC and unstable threshold-dependent behavior), (b) audio-only provides a strong baseline and dominates tasks with predominantly acoustic cues (T04, T05), and (c) fusion offers the largest benefits in tasks where head-motion context disambiguates speech production patterns (T03 and, in ranking terms, T06). This supports the interpretation of IMU as a context signal that regularizes or re-weights acoustic evidence, rather than an independent discriminator.

Table 9 highlights that the “best” method depends on the evaluation target: optimizing ranking performance (AUC) favors gated_early on T03/T06, whereas optimizing a discrete operating point (F1/Accuracy) may select audio_only or early_concat for specific tasks. This motivates reporting both threshold-free and threshold-dependent metrics, and it suggests that task- or cohort-specific threshold calibration could further improve deployment-level performance without changing the backbone architecture.

IMU in isolation is insufficient but useful in fusion. The near-chance performance of the standalone IMU model is expected, given that participants were seated with instructions to maintain a neutral posture, contrasting with high-performance kinematic benchmarks typically obtained from gait or active motor tasks [8,9] gated_early yields the highest pooled AUC and becomes top 1 on T03 and T06, where nuanced motion context complements acoustics. audio_only remains a strong and sometimes superior choice on acoustically dominated tasks (T04, T05), consistent with its top pooled F_1_. The AUC–F_1_ trade-off between fusion and audio-only likely reflects operating-point selection after calibration; under alternative thresholds (e.g., optimizing $t^{★}$ per task), fusion may narrow the F_1_ gap while retaining ranking gains. These results support IMU as a conditional enhancer of audio, with explicit gating improving when and how IMU influences the representation.

## 5. Statistical Analysis and Generalizability

To strengthen generalizability beyond point estimates, we report variability and uncertainty across the outer evaluation folds. Specifically, for each method, we compute the mean and standard deviation (SD) over the five outer folds, together with a 95% confidence interval (CI) for the mean using the Student-*t* distribution. In addition, we assess whether differences between methods and the audio_only baseline are statistically significant using paired Wilcoxon signed-rank tests across folds (two-sided), and we correct for multiple comparisons within each metric using the Holm procedure.

Table 10 summarizes pooled results across all tasks. Among multimodal approaches, gated_early and mid_xattn achieve the highest pooled AUC (0.875 and 0.873, respectively), with comparable F1 (0.771 and 0.763) and Accuracy (0.806 and 0.776). The audio_only baseline remains competitive (AUC 0.865; F1 0.785; Accuracy 0.802). In contrast, imu_only performs near chance in terms of discrimination (AUC 0.497) and yields substantially lower Accuracy (0.458), despite a moderate pooled F1 (0.622), reflecting the limited standalone discriminative utility of head-IMU in this pooled setting.

### 5.1. Statistical Significance Testing (Paired Across Folds)

To test whether observed differences are statistically reliable across outer folds, we compare each method against audio_only using paired Wilcoxon signed-rank tests (two-sided). Given the small number of folds (n=5), this non-parametric paired design avoids normality assumptions. We additionally apply Holm correction for multiple comparisons within each metric.

As shown in Table 11, after Holm correction, none of the pairwise comparisons versus audio_only reach statistical significance for AUC, F1, or Accuracy (all $p_{\mathrm{Holm}}\geq 0.75$). This indicates that, at the fold level, the multimodal variants do not demonstrate a statistically significant improvement over audio_only in the pooled setting, despite modest differences in mean performance.

### 5.2. Implications for Generalizability

Reporting SD and 95% CIs provides a compact characterization of performance variability across outer folds, complementing point estimates and enabling a more transparent assessment of robustness. In our pooled evaluation, performance differences between multimodal fusion methods and the audio_only baseline are small relative to fold-to-fold variability, and the paired significance tests (Holm-corrected) do not support a statistically significant gain over audio_only. These findings motivate future evaluation on larger cohorts and/or additional external sites to increase statistical power and to validate whether multimodal fusion yields consistent improvements beyond the audio baseline under broader acquisition conditions.

## 6. Discussion

This study demonstrates the feasibility and benefits of fusing voice and head motion signals for Parkinson’s disease (PD) assessment in a mixed-reality (MR) environment. We developed a HoloLens 2-based system (DiagNeuro) that records synchronized speech and head inertial data during structured tasks, and we evaluated multiple sensor-fusion strategies.

The principal finding is that combining vocal features with head-mounted IMU data modestly improved PD vs. healthy classification performance over speech alone, while voice features remained the dominant discriminative modality. Specifically, an early-fusion model with a learned gating mechanism achieved the highest overall AUC (≈0.875) (vs. 0.865 for audio_only) and also the best pooled Accuracy (≈0.806; Table 5). Rather than uniformly improving sensitivity, the fusion models exhibit a precision–recall trade-off: mid_xattn yields higher recall (≈0.812) but lower precision (≈0.729), whereas gated_early improves precision (≈0.829) while maintaining competitive recall (≈0.730), confirming that multimodal integration can capture complementary PD markers [41].

This behavior is consistent with the pooled ROC curves (Figure 6), which illustrate the operating regions where fusion provides the clearest separation.

At the same time, a strong voice-only baseline (AUC ≈0.865) reflected the well-known richness of acoustic biomarkers in PD [41]. In our protocol, the head-IMU channel on its own was only weakly discriminative (near-chance AUC), which we attribute primarily to the task and cohort characteristics rather than to hardware limitations. While IMU sensors typically achieve high diagnostic accuracy in gait or active motor tasks [9,17], all recordings were collected during seated speech with a neutral head posture in patients who were predominantly mild–moderate, so the available kinematic signal reflects subtle micromovements rather than overt axial abnormalities. Under these conditions, short IMU segments are expected to be low-contrast between PD and HC. Nevertheless, the same IMU stream yields a consistent, task-dependent gain when fused with audio, especially for tasks where axial–motor differences are more likely to manifest. This suggests that kinesthetic cues are indeed informative but act mainly as a conditional modulator of the much richer acoustic representation. These results support our hypothesis that subtle head-movement patterns (e.g., reduced range or tremor-like activity) contribute additional cues to PD detection when measured concurrently with speech, even though voice signals carry the bulk of the information. We therefore view the weak IMU-only performance as specific to this speech-centric battery and anticipate stronger standalone effects in future work that includes explicit gait/balance or head-movement tasks and a broader range of disease stages.

We also found that fusion efficacy is task-dependent: for highly voice-centric tasks (e.g., reading or rapid syllable repetition), adding IMU yielded no gain or slight degradation, whereas in tasks engaging posture or sustained phonation, the audio–IMU fusion gave measurable improvements. This is consistent with Table 9: audio_only is best on T04/T05, gated_early on T03/T06 (by AUC), and early_concat on T02. This nuanced outcome suggests that an adaptive fusion approach (as implemented by our gating model) is preferable, enabling the model to rely on motion cues only when they are informative.

In summary, DiagNeuro validates the concept of synchronized multimodal MR assessment for PD, showing that dual-channel analysis is not only technically feasible but can modestly enhance detection of PD-related abnormalities compared to single-modality analysis.

Our successful deployment of a 15–20 min MR task battery in over 160 sessions (Table 2) with minimal missing data demonstrates the practicality of this approach in a patient population. These advantages come alongside evidence that AR headsets can produce clinically valid measurements for motor functions: recent studies have shown HoloLens-derived metrics for gait, balance, and functional tests to concur with gold-standard instruments [6]. Our work extends this validity to the concurrent capture of voice and head movement, suggesting that AR devices can be multi-purpose evaluation tools in neurology.

A key question is how our MR-based findings translate to more ubiquitous platforms like smartphones or wearables. On one hand, the HoloLens 2 provided high-fidelity data in a controlled setting—a best-case scenario for digital biomarker capture. Moving to commodity mobile devices will introduce challenges such as variable hardware, environmental noise, and lack of holographic guidance.

A potential translational strategy would be to preserve the standardized task paradigm developed in MR but deliver it through a smartphone app. For example, the app could display written or audio instructions for a sustained phonation, a reading passage, or a brief spoken monologue, similar to our AR tasks. The phone’s microphone can record the speech, while its motion sensors could capture gross movements (if the phone is held in hand or kept in a shirt pocket to approximate upper-body motion). Although this would not exactly replicate head kinematics, it could pick up related signals (hand tremor, body sway or subtle movements as the person speaks). Recent work shows that such multimodal smartphone assessments are feasible and can detect early PD, lending hope that a “lighter” version of DiagNeuro could run on common devices.

In essence, MR-based research guides the design of mobile tools by indicating what to measure and how to standardize the measurement. Our analysis already touched on this by exploring early, intermediate, and late fusion; a mobile app could employ the same fusion strategies in software, even if the sensors differ.

In summary, while direct deployment of DiagNeuro on smartphones is not plug-and-play, the insights gained are transferable. We emphasize that maintaining task consistency is crucial—whether via MR or mobile—because uncontrolled free behavior might not yield the specific biomarkers (e.g., calibrated speech tasks, intentional head movements or steadiness) that a structured exam elicits. Thus, the ideal path to translation is a hybrid—use the phone’s convenience but enforce a protocol akin to an AR session. This could preserve much of the benefit, enabling broader use of multimodal PD assessment outside specialized labs [6].

Despite its promising results, our study has several limitations. First, the data were collected in a supervised MR setting, which may limit generalizability. Participants were aware of being evaluated via an AR headset and were guided through specific tasks. This controlled context is useful for consistency, but real-world conditions differ. In daily life, speech and movement are spontaneous and may be influenced by distractions or varying emotional states. Future work should evaluate our multimodal markers in more naturalistic environments—for example, by having patients use the system at home over longer periods, or by comparing MR-guided results with passive monitoring (like analyzing free speech during phone calls).

Secondly, our cohort consisted of moderate-stage PD patients and age-matched healthy controls, but we did not explicitly stratify by disease severity or phenotype. It remains unclear how early in the disease these voice and head-motion changes become detectable. Prior research indicates that acoustic changes can precede overt motor signs in PD [6], so a worthwhile next step is to test early-stage or even prodromal individuals with our pipeline. The head movement differences in early PD might be very subtle (since axial rigidity and postural impairment typically worsen in later stages), potentially yielding a smaller fusion benefit. Longitudinal studies are also needed: can our synchronized metrics track disease progression or responses to therapy? Repeated MR assessments (for example, every few months) could show whether the multimodal score correlates with clinical changes over time.

Additionally, the contribution of the head IMU signals, while statistically significant in some cases, was relatively modest. Our best fusion model improved AUC by only about 1–2 percentage points over audio-only in pooled analysis. This improvement might not be clinically meaningful on its own. A detailed error analysis would help clarify when and why the fusion helps It is possible that only a subset of PD patients (those with prominent axial symptoms) benefit from the motion features, whereas others do not. In future research, personalized or subgroup models could be explored, where the system adapts to patient-specific symptom profiles (e.g., placing more weight on IMU features for patients with higher axial rigidity or tremor).

Moreover, incorporating other modalities could amplify the gains: our platform could readily be extended with, say, a hand tremor task using the controller or an eye-tracking task, as we have prototyped separately. Fusing more than two modalities (e.g., voice, head motion, and eye movement) is an exciting direction made feasible by MR headsets. However, this also raises the challenge of feature overload and the need for efficient fusion algorithms to avoid noise from less informative channels.

The next point worth discussing is that our task battery focused on speech and did not include overt gait or balance tasks. This was by design (to target hypokinetic dysarthria and head micro-movements), but it means that some cardinal PD features were not probed. A comprehensive AR exam could integrate our voice + head tasks with brief motor tasks (like an on-spot stepping or sway test) to capture a wider spectrum of PD signs. Doing so might increase overall diagnostic accuracy, as suggested by multi-domain smartphone studies.

Relatedly, we did not combine the results across tasks for a subject-level diagnosis in this work—each task episode was classified independently. In practice, a clinician would consider the aggregate of a patient’s performance across multiple tasks. We expect that simple voting or averaging of the per-task outputs would improve stability and reduce false alarms. Future implementations of DiagNeuro can incorporate subject-level decision logic, possibly with learned weighting for each task’s contribution. Lastly, the current system’s hardware (HoloLens 2) may not be easily accessible or tolerable for all patients. The device is relatively heavy and expensive, and wearing it might be uncomfortable for some elderly users or those with neck issues. While our participants generally managed the 15-minute sessions well, a few reported mild fatigue. As AR technology evolves, lighter glasses or even AR contact lenses could alleviate this limitation. In the meantime, careful protocol design (e.g., offering breaks, ensuring proper fit) is important when using such headsets clinically. We also acknowledge that some training is needed for users to get acquainted with MR interaction (like using gaze or voice commands to navigate tasks), though in our experience the learning curve was short. Going forward, usability studies should be conducted to optimize the patient experience—particularly if assessments are to be carried out at home without technical support. To provide a final contextualization of our results, Table 12 summarizes the performance of the DiagNeuro system alongside key unimodal and multimodal benchmarks referenced in this study. This overview confirms our conclusion: although our seated protocol provides lower standalone kinematic discriminability compared to dynamic gait assessments, multimodal fusion effectively fills this gap.

## 7. Conclusions

This work sets the stage for truly multimodal digital biomarkers in Parkinson’s disease by leveraging an immersive MR platform. We demonstrated that synchronized voice and head-motion analysis is feasible and can modestly enhance detection of PD-related deficits, reinforcing the notion that no single sensor tells the whole story. The AR paradigm not only improves data quality through standardization but also opens new avenues for patient engagement and at-home monitoring.

As technology progresses, the gap between specialized MR systems and everyday mobile devices will continue to narrow. Our approach can be seen as a testbed for next-generation digital exams—one that can be iteratively distilled into more portable formats.

We envision a future where a patient might perform a brief multimodal task routine (speaking, moving, looking at targets) guided either by AR glasses or a smartphone app, and receive an immediate, objective report on their motor and speech health. Realizing this vision will require interdisciplinary efforts, validation in larger and more diverse cohorts, and close collaboration with clinicians to ensure that the digital scores map onto meaningful clinical outcomes.

In conclusion, this study validates DiagNeuro, a novel HoloLens 2-based system for the synchronized, multimodal assessment of Parkinson’s disease (PD) using voice and head-motion signals in a mixed-reality (MR) environment. The key finding demonstrates that fusing these two modalities modestly improved PD classification accuracy (reaching a peak AUC of ≈0.875) over the already strong voice-only baseline (AUC ≈0.865). This improvement confirms that head kinematics, even when subtle and acquired during seated speech tasks, provide complementary, task-dependent information that, when integrated via an adaptive fusion mechanism, enhances PD detection. This work successfully establishes the technical feasibility and standardization benefits of the MR platform for capturing dual-channel digital biomarkers. Future research should prioritize the longitudinal validation of these multimodal markers in early-stage and prodromal patients, explore the inclusion of additional modalities or overt motor tasks to amplify the diagnostic gains, and guide the translation of these standardized assessment paradigms to more scalable, ubiquitous mobile platforms for widespread clinical use.

## Figures and Tables

**Table 1:** Representative digital-biomarker and multimodal studies in Parkinson’s disease (PD).

Author(s)	Year	Dataset/Device	Sample Size	Methodology	Performance Metrics	Limitations	Significance
Vásquez-Correa et al. [12]	2019	Speech, handwriting, and gait recordings	PD = 43, HC = 39	Multimodal feature extraction from voice, handwriting, and gait; deep neural network classifier	Accuracy ≈97.6% for PD vs. HC	Very small cohort; no external validation (risk of overfitting)	Early proof-of-concept that combining voice, handwriting, and gait can yield high PD detection accuracy.
Powers et al. [13]	2021	Smartwatch inertial sensors in daily life	PD = 343 (225 with ≥6-month follow-up)	Continuous monitoring of tremor and dyskinesia; wearable-derived scores compared with clinical ratings	Correlations up to ρ≈0.8 with clinical tremor severity; good detection of ON/OFF fluctuations	Focused on tremor/dyskinesia; requires long-term device wear and adherence	Demonstrated feasibility of continuous real-world monitoring of motor fluctuations using wrist-worn IMUs.
Burq et al. [14]	2022	Active smartwatch-based motor tests performed at home	PD = 388 (early-stage; no controls)	Unsupervised remote exam combining tremor, gait, and tapping tasks; sensor features related to MDS-UPDRS scores	Moderate-to-strong correlations with motor ratings (ρ≈0.46–0.70); test–retest ICC ≈0.75–0.96; sensitivity to medication state (Cohen’s *d* up to ≈0.5)	No healthy control group; requires frequent active participation and high compliance	Validated that wearable-based remote exams can consistently quantify PD motor symptoms and detect fluctuations, supporting use as digital endpoints in trials.
van Bergem et al. [9]	2024	AR glasses (HoloLens 2/Magic Leap 2) for gait and balance tests	PD = 22	Head-mounted AR device tracked timing of Timed Up-and-Go (TUG) and Five-Times-Sit-to-Stand (FTSTS); compared AR-derived timings with stopwatch measures	Excellent agreement between AR and clinical timing (bias <4%, ICC ≈0.98) and high test–retest reliability	Small sample; primarily evaluated task durations (limited biomechanical detail)	Showed that AR glasses can validly and reliably capture standard gait/balance metrics in PD, supporting their use in clinical assessments.
Adams et al. [15]	2024	Smartphone and smartwatch motor assessments (baseline PD vs. HC; 12-month PD follow-up)	PD = 82 (untreated, early), HC = 50	In-clinic digital tests plus twice-weekly at-home tasks (gait, tremor, tapping, speech); longitudinal modeling of digital features	Wearable metrics significantly differentiated PD vs. HC and showed measurable progression over 12 months, in some cases more sensitive than clinical scores	Moderate cohort size; data loss when treatment started; relies on regular device use and task completion	Demonstrated that consumer-grade devices can detect subtle motor progression in early PD and provide sensitive digital endpoints.
Illner et al. [16]	2024	Passive smartphone voice calls plus brief prompted reading	iRBD * = 21, early PD = 26, HC = 25	Acoustic feature analysis of real-world call audio and daily reading; machine-learning classification of groups	AUC ≈0.85 for distinguishing high-risk iRBD from controls using combined passive and active speech	Focused on an at-risk iRBD cohort; not yet validated for population-level screening; susceptible to variable call environments	Provided a proof-of-concept that everyday voice calls can act as early digital biomarkers of parkinsonism in prodromal populations.
van Doorn et al. [17]	2025	AR headset 10-m walk test (HoloLens 2)	PD = 20	Head-position tracking to derive gait parameters (step length, cadence, speed) at comfortable and fast speed; compared with reference gait measurements	Strong concurrent validity and excellent reliability (ICC >0.94 for step length and walking speed) across conditions	Short walking bouts; head sensor provides indirect view of leg motion; small cohort	Confirmed that AR glasses can accurately and reliably measure basic gait characteristics in PD, supporting AR-based mobility assessment.
Hemmerling et al. [18]	2025	Mixed-reality Timed Up-and-Go (TUG) dataset from HoloLens 2 IMU	PD and HC, tens of participants per group	Head-IMU features (e.g., turn duration, trunk rotation proxies) and machine-learning classifiers for PD vs. HC discrimination	Best model achieved balanced accuracy ≈81% (F1 ≈0.78) for PD vs. HC classification	Single task and single head-worn sensor; results need validation in free-living or more complex mobility contexts	Demonstrated that MR-based head kinematics alone can yield promising PD vs. HC discrimination, motivating richer multimodal MR pipelines.
Dudek et al. [6]	2025	MR-captured speech and language tasks via HoloLens 2	PD = 21, HC = 36	Standardized mixed-reality voice tasks (sustained vowel, reading, monologue); acoustic and linguistic features; classical ML and deep-learning classifiers	Task-dependent performance with F1 scores up to ≈0.94 for PD vs. HC in rapid syllable tasks and ≈0.90 in story-retelling	Moderate sample; single language and device; focuses on voice modality only (no kinematic fusion)	Showed that MR-based standardized voice capture provides strong PD detection performance and is well-suited for future multimodal MR pipelines.
Lim et al. [19]	2025	Smartphone app measuring voice, finger tapping, and gait	PD = 213, HC = 283 (early PD, ON/OFF medication)	App-guided active tests; features from each modality; single-modality and fused machine-learning models (including SVM) for PD identification	Voice-, tapping-, and gait-only models reached AUROC ≈0.80, 0.74, and 0.76; multimodal fusion improved AUROC to ≈0.82	Requires active test performance and compliance; assessments in semi-controlled settings, not fully passive real-world data	Demonstrated that multimodal smartphone-derived features outperform single-domain models for early PD detection, highlighting the value of integrated digital biomarkers.

* iRBD = idiopathic REM sleep behaviour disorder, often considered a prodromal stage of PD.

**Table 2:** Dataset completeness summary by group.

Group	Participants	Sessions	Audio	Accel	Gyro	Complete
HC	93	464	216	218	218	211
PD	72	358	175	182	181	175

**Table 3:** Task-wise dataset summary per group. The table details demographics (age and sex) alongside the recording inventory: number of sessions, available recordings for each modality (Audio/Accelerometer/Gyroscope), and complete sessions—containing all three synchronized modalities. Clinical means are provided for the PD group. Values are presented as mean (standard deviation).

Task ID	Age,y	Sex (M/F)	Sessions	Modalities	Complete	UPDRS-III	UPDRS-T	H&Y	S&E	MSDSRS	VHI
T02	66.1 (8.7)	22/21	93	44/44/44	43	–	–	–	–	–	–
	62.4 (11.9)	17/16	71	33/36/36	33	1.3 (0.8)	28.7 (15.6)	2.2 (1.0)	0.8 (0.2)	7.3 (1.7)	24.2 (26.5)
T03	66.7 (8.6)	21/20	93	42/44/44	41	–	–	–	–	–	–
	64.4 (11.5)	18/17	71	35/36/36	35	1.4 (1.0)	29.3 (16.3)	2.2 (1.0)	0.7 (0.2)	7.3 (1.7)	25.4 (28.0)
T04	69.2 (7.8)	21/21	93	43/43/43	42	–	–	–	–	–	–
	65.9 (11.7)	17/17	72	34/36/36	34	1.4 (0.9)	31.0 (19.4)	2.3 (1.1)	0.7 (0.2)	7.5 (1.7)	23.6 (26.8)
T05	67.1 (9.8)	22/21	93	44/44/44	43	–	–	–	–	–	–
	65.5 (11.7)	20/17	73	37/38/37	37	1.4 (0.8)	29.7 (17.4)	2.4 (1.1)	0.8 (0.2)	7.2 (1.6)	25.3 (25.5)
T06	63.4 (9.7)	21/21	92	43/43/43	42	–	–	–	–	–	–
	63.9 (12.1)	16/20	71	36/36/36	36	1.5 (0.9)	32.5 (19.2)	2.4 (1.1)	0.7 (0.2)	7.4 (1.7)	21.6 (26.5)

**Group**: 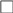 HC (93 participants), 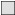 PD (72 participants).

**Table 4:** Fixed training and inference hyperparameters shared across all model variants, with fusion-specific settings.

Hyperparameter	Value
Shared across all variants (audio_only, imu_only, early_concat, gated_early, mid_xattn)	
Audio sampling rate	16 kHz (mono, peak-normalized)
Log-mel features	F=56, $N_{\mathrm{FFT}}=1024$, hop =320 samples, log floor $10^{-10}$
IMU preprocessing	100 Hz resampling; 6 channels (ACC + GYR); median-center + z-score; std floor $10^{-6}$
Windowing/crops	15 s (train: random crop; test: TTA)
TTA	K=3 random crops; logits averaged
Outer CV	5-fold StratifiedGroupKFold (participant-grouped)
Inner validation	3× GroupShuffleSplit (70/30, participant-grouped)
Repeats per outer fold	3 independent runs; calibrated probabilities averaged
Embedding size	H=96
Audio encoder	2×Conv1D over time, kernel = 5, ReLU
IMU encoder	per-timestep MLP 6→96 (Linear–ReLU–Linear)
Positional encoding	sinusoidal, added to embeddings
Temporal backbone (matched)	BiGRU, 1 layer, hidden $H_{\mathrm{gru}}=128$ per direction
Classifier head (matched)	MLP $2H_{\mathrm{gru}}$(=256)→128→1, ReLU, dropout p=0.20
Loss	class-weighted cross-entropy (computed on training fold)
Optimizer	AdamW ($\beta_{1}=0.9,\beta_{2}=0.999,\epsilon =10^{-8}$)
Batch size/epochs	32/max 50
LR/weight decay	$10^{-3}$/$10^{-2}$ (constant LR; no scheduler)
Gradient clipping	max_norm =1.0
Early stopping	smoothed val AUC (last-3 mean), patience =4
Calibration	temperature scaling on inner validation; T∈[0.3,3.0]
Operating threshold	$t^{★}\in \left[0.2,0.8\right]$; fallback $T=1.0,t^{★}=0.5$ if $N_{\mathrm{val}}<20$
Threshold selection	maximize validation F1 on calibrated probabilities
Random seed	42 (splits + training + TTA sampling)
Precision	FP32 (no AMP)
Fusion-specific settings (only where applicable; otherwise N/A)	
early_concat	concatenate $\left[a_{t}∥i_{t}\right]\in R^{2H}$; no extra parameters
gated_early gate	gate MLP 2H(=192)→96→2H, sigmoid; dropout =0.10
mid_xattn attention	MHA with 4 heads, $d_{\mathrm{head}}=24$ (H=96); attn dropout =0.10

**Table 5:** Pooled performance incl. accuracy (means over 5 folds).

Method	AUC	F1	Accuracy	Precision	Recall
gated_early	0.875	0.771	0.806	0.829	0.730
mid_xattn	0.873	0.763	0.776	0.729	0.812
audio_only	0.865	0.785	0.802	0.799	0.785
early_concat	0.847	0.745	0.764	0.758	0.753
imu_only	0.497	0.622	0.458	0.455	0.983

**Table 6:** Per-task AUC (means over 5 folds). Bold marks the best per row.

Task	Imu_Only	Early_Concat	Audio_Only	Mid_Xattn	Gated_Early
T02	0.479	**0.854**	0.823	0.817	0.750
T03	0.435	0.926	0.920	0.911	**0.936**
T04	0.580	0.897	**0.926**	0.893	0.919
T05	0.660	0.759	**0.805**	0.741	0.732
T06	0.579	0.848	0.843	0.825	**0.854**

**Table 7:** Per-task F1 (means over 5 folds). Bold marks the best per row.

Task	Imu_Only	Early_Concat	Audio_Cnly	Mid_Xattn	Gated_Early
T02	0.488	**0.722**	0.710	0.693	0.591
T03	0.497	0.812	0.733	0.803	**0.821**
T04	0.614	0.784	**0.866**	0.746	0.813
T05	0.552	0.657	**0.696**	0.657	0.656
T06	0.536	**0.761**	0.657	0.692	0.663

**Table 8:** Per-task Accuracy (means over 5 folds). Bold marks the best per row.

Task	Imu_Only	Early_Concat	Audio_Only	Mid_Xattn	Gated_Early
T02	0.422	0.732	**0.790**	0.737	0.682
T03	0.461	0.821	0.739	0.808	**0.846**
T04	0.446	0.805	**0.870**	0.716	0.830
T05	0.474	0.651	**0.676**	0.654	0.624
T06	0.528	**0.768**	0.619	0.691	0.692

**Table 9:** Best method per task (means over 5 folds).

Task	Best Method	AUC	F1	Accuracy	Precision	Recall
T02	early_concat	0.854	0.722	0.732	0.744	0.780
T03	gated_early	0.936	0.821	0.846	0.876	0.821
T04	audio_only	0.926	0.866	0.870	0.871	0.871
T05	audio_only	0.805	0.696	0.676	0.618	0.810
T06	gated_early	0.854	0.663	0.692	0.671	0.685

**Table 10:** Pooled performance across all tasks with uncertainty (means over 5 folds; mean ± SD and 95% CI for the mean).

Method	AUC (Mean ± SD) [95% CI]	F1 (Mean ± SD) [95% CI]	Accuracy (Mean ± SD) [95% CI]
gated_early	0.875 ± 0.060 [0.800, 0.949]	0.771 ± 0.070 [0.684, 0.857]	0.806 ± 0.056 [0.736, 0.876]
mid_xattn	0.873 ± 0.055 [0.805, 0.941]	0.763 ± 0.048 [0.704, 0.823]	0.776 ± 0.030 [0.738, 0.813]
audio_only	0.865 ± 0.063 [0.787, 0.943]	0.785 ± 0.054 [0.718, 0.851]	0.802 ± 0.060 [0.728, 0.877]
early_concat	0.847 ± 0.084 [0.743, 0.951]	0.745 ± 0.085 [0.639, 0.851]	0.764 ± 0.095 [0.645, 0.882]
imu_only	0.497 ± 0.045 [0.441, 0.554]	0.622 ± 0.014 [0.604, 0.639]	0.458 ± 0.005 [0.453, 0.464]

**Table 11:** Paired Wilcoxon signed-rank tests vs. audio_only across outer folds, with Holm correction (two-sided).

Comparison	Metric	p_Raw	p_Holm
imu_only vs. audio_only	AUC	0.0625	0.75
early_concat vs. audio_only	AUC	0.3125	1.00
mid_xattn vs. audio_only	AUC	0.8125	1.00
gated_early vs. audio_only	AUC	0.6250	1.00
imu_only vs. audio_only	F1	0.0625	0.75
early_concat vs. audio_only	F1	0.1875	1.00
mid_xattn vs. audio_only	F1	1.0000	1.00
gated_early vs. audio_only	F1	0.6250	1.00
imu_only vs. audio_only	Accuracy	0.0625	0.75
early_concat vs. audio_only	Accuracy	0.1875	1.00
mid_xattn vs. audio_only	Accuracy	0.6250	1.00
gated_early vs. audio_only	Accuracy	1.0000	1.00

**Table 12:** Comparison of the proposed method with related studies using unimodal (Voice, IMU) and multimodal approaches.

Study	Modality	Task/Sensor	Metrics	Result (Approx.)
Vasquez-Correa et al. [12]	Voice	Phonation (Studio mic)	Accuracy	85–97%
Miller Koop et al. [8]	Kinematic	Gait (HoloLens 2)	Validity	>0.90
Powers et al. [13]	Kinematic	Motor fluctuation (Wearable)	Correlation	r≈0.7
Adams et al. [15]	Multimodal	Watch + Phone (Active tests)	Accuracy	≈81%
Current Study	Voice (Unimodal)	Speech Battery (AR)	AUC	0.865
Current Study	Head IMU (Unimodal)	Sedentary Motion (AR)	AUC	0.497
Current Study	Fusion	Voice + Head IMU (AR)	AUC	0.875

## Data Availability

Due to ethical restrictions our data collected from Microsoft Hololens 2 Head Mounted Display will be shared upon request.
